# Retroviral Env Glycoprotein Trafficking and Incorporation into Virions

**DOI:** 10.1155/2012/682850

**Published:** 2012-07-02

**Authors:** Tsutomu Murakami

**Affiliations:** AIDS Research Center, National Institute of Infectious Diseases, Toyama 1-23-1, Shinjuku, Tokyo 162-8640, Japan

## Abstract

Together with the Gag protein, the Env glycoprotein is a major retroviral structural protein and is essential for forming infectious virus particles. Env is synthesized, processed, and transported to certain microdomains at the plasma membrane and takes advantage of the same host machinery for its trafficking as that used by cellular glycoproteins. Incorporation of Env into progeny virions is probably mediated by the interaction between Env and Gag, in some cases with the additional involvement of certain host factors. Although several general models have been proposed to explain the incorporation of retroviral Env glycoproteins into virions, the actual mechanism for this process is still unclear, partly because structural data on the Env protein cytoplasmic tail is lacking. This paper presents the current understanding of the synthesis, trafficking, and virion incorporation of retroviral Env proteins.

## 1. Introduction

**Figure 1:**
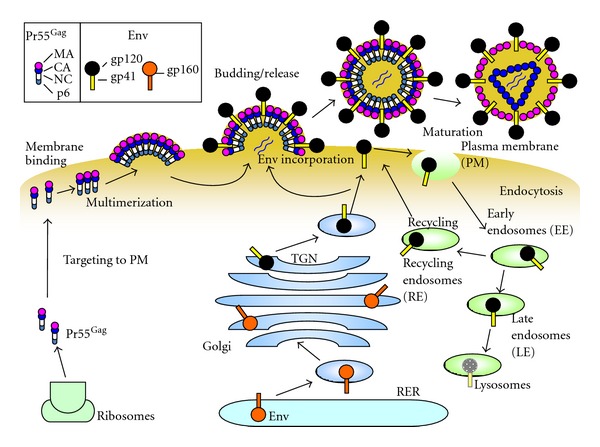
Synthesis and trafficking of HIV-1 Gag and Env proteins. Precursor Gag (Pr55^Gag^) (left) is synthesized on cytosolic ribosomes and traffics to the plasma membrane (PM), where it forms multimers (middle). Env is synthesized as the gp160 precursor, and undergoes glycosylation and oligomerization in the RER. Oligomerized gp160 is transported to the Golgi and the TGN, where it is processed into the surface glycoprotein gp120 and the transmembrane glycoprotein gp41 by cellular enzymes. The gp120/gp41 complexes are transported through the secretory pathway to the PM and are incorporated into virus particles (middle). At the PM, most of the Env protein is endocytosed into early endosomes (EE), which mature into late endosomes (LE) and then into lysosomes for Env degradation (right). However, some Env proteins are recycled to the PM through recycling endosomes (RE). During and after virus release, processing of Pr55^Gag^ by virus proteases yields mature virions. The protein domains of Pr55^Gag^ and Env are illustrated in the insert at the top left. The illustration was adapted from Checkley et al. with permission from Elsevier [23].

All replication-competent retroviruses encode genes for three major proteins: Gag, Pol, and Env. Complex retroviruses, such as human immunodeficiency virus type 1 (HIV-1), encode additional regulatory and accessory proteins required for efficient replication in host cell or the infected host organism. Gag, an essential retroviral protein, is necessary and sufficient for the assembly, budding, and release of virus-like particles (VLPs) in all types of retroviruses except the spumaviruses. Gag is synthesized on cytosolic ribosomes and is assembled as a polyprotein precursor. During and/or shortly after budding and release, the polyprotein is cleaved into several domains by the viral protease (Figure 1) as reviewed in [1–3]. The major domains of the precursor Gag are the matrix (MA), capsid (CA), and nucleocapsid (NC). The primary role of the N-terminal MA domain is targeting of the Gag precursor protein to the site of assembly, typically the plasma membrane (PM). In general, electrostatic interactions between basic amino acid residues in MA and the acidic inner leaflet of the PM are important for Gag-membrane targeting [4, 5]. In the case of HIV-1, the N-terminal myristate group and a cluster of basic residues in the MA domain of the HIV-1 Gag that interacts with phosphatidylinositol-4,5-bisphosphate (PI(4,5)P_2_) together target the Gag precursor Pr55^Gag^ to the PM [6, 7]. Although the Gag-membrane targeting of both murine leukemia virus (MLV) and Mason-Pfizer monkey virus (MPMV) is also affected by PI(4,5)P_2_ modulation [8, 9], it has been reported that the membrane targeting of Rous sarcoma virus (RSV) and human T-lymphotropic virus type 1 (HTLV-1) is largely independent of PI(4,5)P_2_ [10, 11]. The MA domain also plays a role in the incorporation of the Env glycoprotein into virions. The CA domain is important for Gag-Gag interactions during virus assembly and constitutes the outer part of the viral core after Gag processing by the viral protease [12–14]. NC is the primary nucleic acid binding domain of Gag. This small, basic domain is responsible for the binding and incorporation of the viral RNA genome into virions, which is mediated by Gag interactions with genomic RNA.

Gag proteins are synthesized and transported to the PM. Many studies demonstrate that the major site of HIV-1 assembly is the PM [15–18], although late endosomes could be a platform for virus assembly under specific conditions [19]. In primary macrophages, HIV-1 has been shown to assemble in endosomal vesicles. However, studies have recently suggested that the above vesicles are not late endosomes but rather membrane invaginations connected to the PM [20–22].

In addition to Gag, the other major structural retroviral protein is the Env glycoprotein. Env proteins are required for virus entry into target cells and are thus essential for forming infectious retroviral particles. In this paper, we discuss current knowledge about the biosynthesis, intracellular trafficking, and virion incorporation of retroviral Env proteins, as well as the membrane microdomains involved in virus assembly and/or transfer. Most of this information was obtained from studies on HIV-1.

## 2. Env Biosynthesis and Trafficking to the Plasma Membrane

Retroviral Env glycoproteins are synthesized from a spliced form of the viral genomic RNA as reviewed in [23–25] (Figure 1). Translation of the Env protein occurs on ribosomes bound to the endoplasmic reticulum (ER) and starts with the leader sequence, which contains a small, N-terminal hydrophobic signal peptide. The Env protein is cotranslationally inserted into the lumen of the rough ER. In the ER, the leader sequence is removed by cellular signal peptidases. In addition, Env polypeptides are N- and O-glycosylated and subsequently trimmed [26, 27]. The number and location of glycosylated residues varies broadly among retroviruses. The hydrophobic transmembrane (TM) domain prevents Env proteins from being fully released into the lumen of the ER [28, 29]. The amino acid sequence following the TM is referred to as the cytoplasmic tail (CT), which varies from 30 to around 150 residues, depending on the virus. Env proteins are folded and assembled into oligomers in the RER. Retroviral Env proteins form trimers [30–33]. The HIV-1 accessory protein Vpu binds to the CD4 receptor through its cytoplasmic domain and downregulates the receptor by transporting it to the proteasome for degradation, thereby preventing premature interactions between Env and its receptor [34–36].

In the Golgi, cleavage of the retroviral Env precursor occurs at a polybasic (e.g., K/R-X-K/R-R) motif by cellular proteases such as furin or closely related enzymes probably within or near the trans-Golgi network (TGN) [37–43]. For HIV-1, the surface glycoprotein gp120 and the TM glycoprotein gp41, which bind together noncovalently, are both formed from the same precursor protein, gp160. Gp160 processing is essential for the activation of Env fusogenicity and virus infectivity [38, 42, 44–46]. Similarly, cleavage of Env is also essential for membrane fusion and virus infectivity in MLV [39, 47–50], in RSV [51, 52], and in mouse mammary tumor virus (MMTV) [53]. A recent report showed that cleavage of MLV Env by furin also plays an important role in Env intracellular trafficking and incorporation [54]. Although most retroviral Env proteins including that of HIV-1 are associated with intracellular membranes [55–57], at least part of the gp120/gp41 trimer complex traffics through the secretory pathway to the PM. It has been suggested that AP-1, one of adaptor proteins for clathrin-coated vesicle formation, is involved in the correct sorting of HIV-1 Env from the TGN to the PM, [58, 59]. It has been reported that intracellular CTLA-4-containing secretory granules are involved in the trafficking of HIV-1 Env to the PM although the subsequent trafficking of Env after the Golgi is not well understood [60].

After reaching the PM, like those of other lentiviruses, HIV-1 Env undergoes rapid endocytosis, which is mediated by the interaction between the *μ*2 subunit of the clathrin adaptor AP-2 and a membrane-proximal, Tyr-based motif (YxxL) in the gp41 CT [58, 61, 62]. Although some of the endocytosed Env is recycled back to the PM, most retroviral Env is associated with intracellular membranes [63, 64]. The level of gp120-gp41 oligomers on HIV-1 virions is relatively low [33]. Maintaining low levels of Env at the cell surface allows the infected cells to evade the host immune response and to avoid induction of Env-mediated apoptosis. Gammaretroviruses such as MLV and MPMV also have dileucine- and Tyr-based motifs in their Env CT. These motifs are important to regulate intracellular trafficking of Env of both retroviruses via interactions with clathrin adaptors [65, 66].

As for pseudotyping of gammaretroviruses, it has been reported that the feline endogenous retrovirus RD114 Env does not allow pseudotyping with viral cores from lentiviruses such as SIV, whereas the RD114 Env is incorporated into MLV virions [67–69]. Intracellular trafficking of Gag and Env was examined using a set of chimeric viruses between MLV and RD114 [57]. Interestingly, it was found that the RD114 Env was mainly localized along the secretory pathway, whereas the MLV Env was mostly localized in endosomes, and that intracellular localization was dependent on specific motifs in the Env CT [57]. In addition, subsequent work revealed that an acidic cluster in the RD114 Env CT regulates assembly of not only the RD114 Env but also the MLV Env through the interaction with a host factor, phosphofurin acidic-cluster-sorting protein 1 [66].

## 3. Env Incorporation into Virions

**Figure 2:**
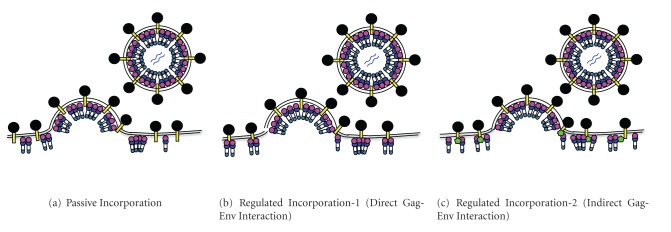
Proposed models for Env incorporation. (a) The passive incorporation model assumes no interaction between Gag and Env. (b) In the first regulated incorporation model, a direct interaction between the MA domain of Gag and the CT domain of Env occurs during Env incorporation. (c) In the second regulated incorporation model, Gag and Env interact indirectly through a bridging protein (green pentagon) that binds to both proteins. The color scheme for Gag and Env is the same as that in Figure 1. The illustration was adapted from Checkley et al. with permission from Elsevier [23].

Several models have been proposed for the incorporation of retroviral Env glycoprotein into virions as reviewed in [23, 70] (Figure 2).

### 3.1. Passive Incorporation

Passive incorporation is the simplest model for the incorporation of Env proteins into virus particles (Figure 2(a)). There are several lines of evidence supporting this model.

First, viral pseudotyping with a foreign glycoprotein can occur easily in many cases although there are some exceptions, one of which is the exclusion of HIV-1 or SIV Env with the long CT from most retrovirus cores [70]. With respect to HIV-1, the virus can be pseudotyped with Env glycoproteins not only from several other retroviruses but also with those from other virus families such as ortho (para) myxoviruses and flaviviruses [71–84].

Second, retroviruses allow passive incorporation of host membrane proteins into virus particles [85–87]. Most cellular proteins are incorporated into the retrovirus envelope without significant sorting [88, 89].

Finally, in the case of HIV-1, several studies have demonstrated that the gp41 CT can be removed without affecting incorporation of the Env into virions, although this has been shown to occur only for some laboratory cell lines such as HeLa or 293T [90–94].

### 3.2. Regulated Incorporation through Direct Gag-Env Interactions

Although several lines of evidence support the passive incorporation model for retroviral Env, there is much evidence indicating that Env incorporation into virions is regulated by direct interactions between Gag and Env proteins (Figure 2(b)). Although removal of the gp41 CT sequence of HIV-1 has little effect on Env incorporation in some cell types, as described above, smaller deletions in CT regions cause severe defects in Env incorporation [95–100]. The MA domain of Gag has been shown to be important for Env incorporation into virions [91, 92, 101, 102]. The defect in Env incorporation caused by deletion of the gp41 CT is reversed by several MA mutations, indicating that an interaction between Env and the MA domain of Gag is required for incorporation of full-length Env into virions, at least in the case of HIV-1 [93, 98].

More evidence for direct Gag-Env interaction comes from the finding that HIV-1 Env directs Gag budding to the basolateral surface of polarized epithelial Madin-Darby canine kidney (MDCK) cells through the CT of HIV-1 Env, whereas Gag alone buds in a nonpolarized fashion [103–106]. The Tyr-based motif in the gp41 CT is also utilized in polarized budding of HIV-1 in lymphocytes [107]. Surprisingly, the polarized budding of HIV-1 in MDCK cells could also be promoted by MLV and HTLV-1 Env through their CT [108]. It also has been reported that coexpression of Pr55^Gag^ inhibits endocytosis of HIV-1 Env through its interaction with the gp41 CT [63]. Another example of the specific Gag-Env interactions was demonstrated using Gag and Env proteins of MLV and HIV-1 in rat neurons [109]. Similarly, MLV Env is preferentially recruited onto MLV Gag through its CT domain in the presence of both MLV and HIV-1 cores although the authors also show an alternative mechanism by which the recruitment to HIV-1 budding sites is independent of the CT domain of MLV Env [110]. Furthermore, RSV Env is exclusively recruited to RSV budding sites through its CT, suggesting that the interaction between Env and Gag is direct in the case of this avian retrovirus [111].

In addition to the circumstantial evidence discussed above, some biochemical data suggest a direct interaction between Gag and Env. *In vitro* binding between MA and a gp41 CT-GST fusion protein has been reported for both HIV-1 and SIV [112, 113]. Peptides corresponding to a large central domain of gp41 CT inhibited the capture of membrane-free Pr55^Gag^ with an anti-p24 antibody [114]. In addition, a stable, detergent-resistant gp41-Pr55^Gag^ interaction was detected in immature HIV-1 virions. The retention of gp41 in detergent-treated virions is dependent on the CT region, suggesting a direct or indirect interaction between Pr55^Gag^ and gp41 [115, 116].

### 3.3. Regulated Incorporation through Indirect Gag-Env Interactions

In the third model, it is assumed that host cellular factors (mostly proteins) play a role in bridging Gag and Env in virus-infected cells (Figure 2(c)). Several host factors have been reported to bind to Gag and/or Env of HIV-1 or SIV however, only a couple of host factors were shown to be required for Env incorporation and/or viral replication.

The 47-kDa tail-interacting protein (TIP47) has been reported to bridge Gag and Env, allowing efficient Env incorporation in HIV-1 [117, 118]. The same group also showed that both the WE motif near the N-terminus of the MA domain and the YW motif in the gp41 CT domain are important for interactions between Gag or Env and TIP47 [118]. In a subsequent paper, the same group showed that mutations in either the WE motif of MA or the YW motif in the gp41 CT caused defects in virus replication in primary monocyte-derived macrophages [119]. Although this finding of an important role for TIP47 in Env incorporation in HIV-1 has received much attention from retrovirologists, no confirmatory data have been published by other researchers in this field.

Human discs large protein (hDlg1) has been reported to interact with the CT of HTLV-1 Env and to colocalize with both Env and Gag in virus-infected cells [120]. Subsequent work demonstrated that Dlg1 also binds HIV-1 Gag and that the expression level of Dlg1 is inversely correlated with HIV-1 Env expression and incorporation levels of the Env proteins, although the mechanism behind this phenomena needs to be investigated [121].

Prenylated Rab acceptor 1 (PRA1), which was identified as a Rab regulatory protein, was reported to be a binding partner for the SIV gp41 CT in a mammalian yeast two-hybrid (Y2H) assay [122]. Although colocalization of PRA1 and SIV Env was observed, changes in the endogenous levels of PRA1 did not affect virus production, Env incorporation, or infectivity of SIV or HIV-1 [123].

A Prohibitin 1/Prohibitin 2 (Phb1/Phb2) heterodimer was identified as a binding partner of the gp41 CT of HIV-1 using human T-cell lines and tandem affinity chromatography [124]. Phb1 and Phb2 are members of the prohibitin superfamily of proteins, which are localized to several cellular compartments such as the mitochondria, nucleus, and the PM [125, 126]. Gp41 CT mutants, in which binding to Phb1/Phb2 is disrupted, could replicate well in permissive cell types such as MT-4, but could not replicate efficiently in nonpermissive H9 cells [124]. Further analysis is necessary to elucidate the mechanism by which these proteins regulate virus replication through interactions with Env.

Luman, a transcription factor that is mainly localized to the ER, was found to interact with the gp41 CT of HIV-1 in a Y2H screen using a cDNA library from human peripheral blood lymphocytes (PBL) [127]. Overexpression of a constitutively active form of this protein reduced the intracellular levels of Gag and Env, leading to a decrease in virus release. The mechanism for this negative effect on virus assembly involves Luman binding to Tat, which decreases Tat-medicated transcription [127].

By using a Y2H screen with human cDNA libraries, p115-RhoGEF, an activator of Rho GTPase, was found to interact with the gp41 CT through its C-terminal regulatory domain [128]. The gp41 mutants that lost the ability to bind p115 showed impaired replication kinetics in T-cell lines such as SupT1, H9, and Jurkat, suggesting that the gp41 CT could modulate the activity of p115-RhoGEF to support virus replication [128].

In addition to the host factors described above, calmodulin [129–132] and *α*-catenin [133–135] have been reported to interact with HIV-1 and/or SIV. However, their roles in virus replication, especially with respect to the Env functions of both proteins, have not been clearly elucidated.

## 4. Membrane Microdomains

Regardless of whether direct or indirect interactions between retroviral Gag and Env proteins are required for Env incorporation into virions, a great deal of experimental evidence suggests that retroviruses assemble and bud from “membrane microdomains.” The most well-known microdomains are “lipid raft(s),” which are enriched in cholesterol and sphingolipids [136, 137]. Lipid rafts are widely thought to function as a platform for the assembly of protein complexes and to allow various biological processes such as cellular transport and signal transduction to proceed efficiently [138, 139]. Lipid rafts are reportedly used as assembly platforms or entry scaffolds in the replication of enveloped viruses such as retroviruses [140–146]. The association of Gag/Env with lipid rafts is important for the regulation of Env incorporation and pseudotyping [143, 144, 147, 148]. Evidence that both the HIV-1 Pr55^Gag^ and Env proteins are preferentially localized to lipid rafts comes from biochemical studies as well as direct observations by microscopy [142, 149, 150].

Another membrane microdomain for retrovirus assembly is the “tetraspanin-enriched microdomain (TEM)” [151–154]. Tetraspanins are a superfamily of cell surface proteins that are ubiquitously expressed in mammalian cells. TEMs also act as platforms for signal transduction and immune responses. TEMs have been reported to be involved in the assembly and release of not only HIV-1, but also HTLV-1 and HCV [155]. When both HIV-1 and influenza virus were produced in the same cell, only HIV-1 colocalized with the TEM marker, and its release was inhibited by an anti-CD9 Ab, which led to extensive aggregation of tetraspanins [156]. Analysis of dynamics of both lipid rafts and TEMs by quantitative microscopy has revealed that components of both lipid rafts and TEMs are recruited during viral assembly to create a new microdomain that is different from preexisting membrane microdomains [153, 157].

There have been three recent reports in which both pseudotyping and microdomain issues were discussed. In the first paper, the authors examined HIV-1 assembly under conditions where the Env proteins of HIV-1 and Ebola virus were coexpressed with HIV-1 Gag in the same cell [158]. They found that infectious HIV-1 virions were released with both types of Env proteins. Interestingly, however, the virions contained either HIV-1 Env or Ebola virus glycoprotein (GP), but not both Env proteins within a single virion. These results suggest that HIV-1 Env and Ebola virus GP localized to distinct microdomains on the surface of the same cell [158]. In the second paper, the subcellular localization of Gag and Env proteins was investigated using a combination of three different retroviral Env proteins (RSV Env, MLV Env, or vesicular stomatitis virus (VSV) G) and two different Gag proteins (RSV or HIV-1) [111]. Both VSV-G and MLV Env were redistributed to the virus budding sites when coexpressed with HIV-1 or RSV Gag. In contrast, RSV Env was mostly transported to RSV budding sites. A subsequent paper from the same group showed that the CT of MLV is not required for recruitment of MLV Env to HIV-1 budding sites, suggesting that there are no specific interactions between MLV Env and HIV-1 Gag [110]. Collectively, these results also suggest that retroviral Env glycoproteins are not recruited to preexisting membrane platforms but rather that they are actively recruited to newly formed microdomains on the cell surface [111].

 Human retroviruses such as HIV-1 and HTLV-1 spread more efficiently between target T cells by cell-cell infection than by cell-free infection [159, 160]. Sattentau et al. proposed, in analogy to the “immunological synapse”, the “virological synapse (VS)” as a point of contact between virus-infected cells and uninfected cells [161, 162]. The molecular mechanisms of retroviral VS formation are as follows. (1) With respect to HIV-1 T-cell VS, initial contact between virus-infected cells and uninfected cells occurs through gp120-CD4 binding. Subsequent interactions between integrins and ICAMs enforce and maintain the stability of these junctions. (2) The gp120-CD4 interaction recruits CD4, coreceptors such as CXCR or CCR5, adhesion molecules, and filamentous actin into the synaptic area. (3) The cellular secretory machinery and microtubule organizing centers (MTOC) are polarized towards the HIV-1 assembly sites at the PM to form the VS. It has been reported that a so-called microsynapse formed by nanotubes between virus-infected cells and uninfected cells is also involved in cell-cell infection of HIV-1 [84, 163]. In cell-cell transfer of HTLV-1-infected cells, an extracellular matrix structure referred to as the “viral biofilm” was proposed as an alternative to the VS [164]. In addition to HIV-1 and HTLV-1, the spread of MLV between fibroblasts also occurs via the VS [165, 166]. It is noteworthy that assembly of MLV is directed towards cell-cell contact sites through the interaction of the CT of MLV Env with Gag [167, 168]. Although the concept of cell-cell infection through the VS is now well appreciated, the detailed molecular mechanism of VS assembly and its relevance to viral spread *in vivo* will require further elucidation through the use of more advanced techniques.

## 5. Conclusions and Perspectives

Incorporation of Env glycoproteins into virions is crucial for producing infectious retroviral particles. Although this paper has introduced several experimental models for retroviral Env trafficking and/or incorporation, the correct mechanism for this process is still unclear. The following questions must be clearly addressed to not only gain a better understanding of this complex biological process, but also to develop new antiretroviral compounds that target Env incorporation.

1. What are the structures of the CTs of retroviral Env proteins? The answers for this question will give useful information on elucidating a role of the Env CTs in the Env trafficking and/or incorporation in virus-infected cells.
2. What host factor(s) are necessary for the retroviral Env trafficking and/or incorporation into virions?
3. Where and how Env and Gag proteins of retroviruses are recruited to the assembly sites in order to form infectious virus particles?

## References

[1] Bieniasz PD (2009). The cell biology of HIV-1 virion genesis. *Cell Host and Microbe*.

[2] Freed EO (1998). HIV-1 Gag proteins: diverse functions in the virus life cycle. *Virology*.

[3] Swanstrom R, Wills JW (1997). *Synthesis, Assembly, and Processing of Viral Proteins*.

[4] Dalton AK, Ako-Adjei D, Murray PS, Murray D, Vogt VM (2007). Electrostatic interactions drive membrane association of the human immunodeficiency virus type 1 Gag MA domain. *Journal of Virology*.

[5] Dalton AK, Murray PS, Murray D, Vogt VM (2005). Biochemical characterization of Rous sarcoma virus MA protein interaction with membranes. *Journal of Virology*.

[6] Ono A, Ablan SD, Lockett SJ, Nagashima K, Freed EO (2004). Phosphatidylinositol (4,5) bisphosphate regulates HIV-1 Gag targeting to the plasma membrane. *Proceedings of the National Academy of Sciences of the United States of America*.

[7] Saad JS, Miller J, Tai J, Kim A, Ghanam RH, Summers MF (2006). Structural basis for targeting HIV-1 Gag proteins to the plasma membrane for virus assembly. *Proceedings of the National Academy of Sciences of the United States of America*.

[8] Chan R, Uchil PD, Jin J (2008). Retroviruses human immunodeficiency virus and murine leukemia virus are enriched in phosphoinositides. *Journal of Virology*.

[9] Stansell E, Apkarian R, Haubova S, Diehl WE, Tytler EM, Hunter E (2007). Basic residues in the Mason-Pfizer monkey virus gag matrix domain regulate intracellular trafficking and capsid-membrane interactions. *Journal of Virology*.

[10] Chan J, Dick RA, Vogt VM (2011). Rous sarcoma virus gag has no specific requirement for phosphatidylinositol-(4, 5)-bisphosphate for plasma membrane association in vivo or for liposome interaction in vitro. *Journal of Virology*.

[11] Inlora J, Chukkapalli V, Derse D, Ono A (2011). Gag localization and virus-like particle release mediated by the matrix domain of human T-lymphotropic virus type 1 gag are less dependent on phosphatidylinositol-(4,5)-bisphosphate than those mediated by the matrix domain of HIV-1 gag. *Journal of Virology*.

[12] de Marco A, Davey NE, Ulbrich P (2010). Conserved and variable features of Gag structure and arrangement in immature retrovirus particles. *Journal of Virology*.

[13] Mortuza GB, Haire LF, Stevens A, Smerdon SJ, Stoye JP, Taylor IA (2004). High-resolution structure of a retroviral capsid hexameric amino-terminal domain. *Nature*.

[14] Pornillos O, Ganser-Pornillos BK, Kelly BN (2009). X-ray structures of the hexameric building block of the HIV capsid. *Cell*.

[15] Finzi A, Orthwein A, Mercier J, Cohen EA (2007). Productive human immunodeficiency virus type 1 assembly takes place at the plasma membrane. *Journal of Virology*.

[16] Ivanchenko S, Godinez WJ, Lampe M (2009). Dynamics of HIV-1 assembly and release. *PLoS Pathogens*.

[17] Jouvenet N, Bieniasz PD, Simon SM (2008). Imaging the biogenesis of individual HIV-1 virions in live cells. *Nature*.

[18] Ono A (2010). Relationships between plasma membrane microdomains and HIV-1 assembly. *Biology of the Cell*.

[19] Joshi A, Ablan SD, Soheilian F, Nagashima K, Freed EO (2009). Evidence that productive human immunodeficiency virus type 1 assembly can occur in an intracellular compartment. *Journal of Virology*.

[20] Bennett AE, Narayan K, Shi D (2009). Ion-abrasion scanning electron microscopy reveals surface-connected tubular conduits in HIV-infected macrophages. *PLoS Pathogens*.

[21] Deneka M, Pelchen-Matthews A, Byland R, Ruiz-Mateos E, Marsh M (2007). In macrophages, HIV-1 assembles into an intracellular plasma membrane domain containing the tetraspanins CD81, CD9, and CD53. *Journal of Cell Biology*.

[22] Welsch S, Keppler OT, Habermann A, Allespach I, Krijnse-Locker J, Kräusslich HG (2007). HIV-1 buds predominantly at the plasma membrane of primary human macrophages. *PLoS Pathogens*.

[23] Checkley MA, Luttge BG, Freed EO 2011 HIV-1 envelope glycoprotein biosynthesis, trafficking, and incorporation. *Journal of Molecular Biology*.

[24] Freed EO, Martin MA (1995). The role of human immunodeficiency virus type 1 envelope glycoproteins in virus infection. *Journal of Biological Chemistry*.

[25] Hunter E, Swanstrom R (1990). Retrovirus envelope glycoproteins. *Current Topics in Microbiology and Immunology*.

[26] Bernstein HB, Tucker SP, Hunter E, Schutzbach JS, Compans RW (1994). Human immunodeficiency virus type 1 envelope glycoprotein is modified by O-linked oligosaccharides. *Journal of Virology*.

[27] Leonard CK, Spellman MW, Riddle L, Harris RJ, Thomas JN, Gregory TJ (1990). Assignment of intrachain disulfide bonds and characterization of potential glycosylation sites of the type 1 recombinant human immunodeficiency virus envelope glycoprotein (gp120) expressed in Chinese hamster ovary cells. *Journal of Biological Chemistry*.

[28] Berman PW, Nunes WM, Haffar OK (1988). Expression of membrane-associated and secreted variants of gp160 of human immunodeficiency virus type 1 in vitro and in continuous cell lines. *Journal of Virology*.

[29] Haffar OK, Dowbenko DJ, Berman PW (1988). Topogenic analysis of the human immunodeficiency virus type 1 envelope glycoprotein, gp160, in microsomal membranes. *Journal of Cell Biology*.

[30] Center RJ, Schuck P, Leapman RD (2001). Oligomeric structure of virion-associated and soluble forms of the simian immunodeficiency virus envelope protein in the prefusion activated conformation. *Proceedings of the National Academy of Sciences of the United States of America*.

[31] Förster F, Medalia O, Zauberman N, Baumeister W, Fass D (2005). Retrovirus envelope protein complex structure in situ studied by cryo-electron tomography. *Proceedings of the National Academy of Sciences of the United States of America*.

[32] Wilk T, de Haas F, Wagner A (2000). The intact retroviral Env glycoprotein of human foamy virus is a trimer. *Journal of Virology*.

[33] Zhu P, Chertova E, Bess JW (2003). Electron tomography analysis of envelope glycoprotein trimers on HIV and simian immunodeficiency virus virions. *Proceedings of the National Academy of Sciences of the United States of America*.

[34] Fujita K, Omura S, Silver J (1997). Rapid degradation of CD4 in cells expressing human immunodeficiency virus type 1 Env and Vpu is blocked by proteasome inhibitors. *Journal of General Virology*.

[35] Margottin F, Bour SP, Durand H (1998). A novel human WD protein, h-*β*TrCP, that interacts with HIV-1 Vpu connects CD4 to the ER degradation pathway through an F-box motif. *Molecular Cell*.

[36] Schubert U, Antón LC, Bačík I (1998). CD4 glycoprotein degradation induced by human immunodeficiency virus type 1 Vpu protein requires the function of proteasomes and the ubiquitin- conjugating pathway. *Journal of Virology*.

[37] Bedgood RM, Stallcup MR (1992). A novel intermediate in processing of murine leukemia virus envelope glycoproteins. Proteolytic cleavage in the late Golgi region. *Journal of Biological Chemistry*.

[38] Freed EO, Myers DJ, Risser R (1989). Mutational analysis of the cleavage sequence of the human immunodeficiency virus type 1 envelope glycoprotein precursor gp160. *Journal of Virology*.

[39] Freed EO, Risser R (1987). The role of envelope glycoprotein processing in murine leukemia virus infection. *Journal of Virology*.

[40] Geiselhart V, Bastone P, Kempf T, Schnölzer M, Löchelt M (2004). Furin-mediated cleavage of the feline foamy virus Env leader protein. *Journal of Virology*.

[41] Hallenberger S, Bosch V, Angliker H, Shaw E, Klenk HD, Garten W (1992). Inhibition of furin-mediated cleavage activation of HIV-1 glycoprotein gp160. *Nature*.

[42] McCune JM, Rabin LB, Feinberg MB (1988). Endoproteolytic cleavage of gp160 is required for the activation of human immunodeficiency virus. *Cell*.

[43] Stein BS, Engleman EG (1990). Intracellular processing of the gp160 HIV-1 envelope precursor. Endoproteolytic cleavage occurs in a cis or medial compartment of the Golgi complex. *Journal of Biological Chemistry*.

[44] Bosch V, Pawlita M (1990). Mutational analysis of the human immunodeficiency virus type 1 env gene product proteolytic cleavage site. *Journal of Virology*.

[45] Dubay JW, Dubay SR, Shin HJ, Hunter E (1995). Analysis of the cleavage site of the human immunodeficiency virus type 1 glycoprotein: requirement of precursor cleavage for glycoprotein incorporation. *Journal of Virology*.

[46] Guo HG, Veronese FM, Tschachler E (1990). Characterization of an HIV-1 point mutant blocked in envelope glycoprotein cleavage. *Virology*.

[47] Famulari NG, Jelalian K (1979). Cell surface expression of the env gene polyprotein of dual-tropic mink cell focus-forming murine leukemia virus. *Journal of Virology*.

[48] Granowitz C, Colicelli J, Goff SP (1991). Analysis of mutations in the envelope gene of Moloney murine leukemia virus: separation of infectivity from superinfection resistance. *Virology*.

[49] Machida CA, Kabat D (1982). Role of partial proteolysis in processing murine leukemia virus membrane envelope glycoproteins to the cell surface. A viral mutant with uncleaved glycoprotein. *Journal of Biological Chemistry*.

[50] Zavorotinskaya T, Albritton LM (1999). Failure to cleave murine leukemia virus envelope protein does not preclude its incorporation in virions and productive virus-receptor interaction. *Journal of Virology*.

[51] Dong J, Dubay JW, Perez LG, Hunter E (1992). Mutations within the proteolytic cleavage site of the Rous sarcoma virus glycoprotein define a requirement for dibasic residues for intracellular cleavage. *Journal of Virology*.

[52] Perez LG, Hunter E (1987). Mutations within the proteolytic cleavage site of the Rous sarcoma virus glycoprotein that block processing to gp85 and gp37. *Journal of Virology*.

[53] Goodman LJ, Kain SR, Firestone GL (1993). Trafficking of wild-type and an endoproteolytic-site mutant of the mouse mammary tumor virus glycoprotein. *Journal of Biological Chemistry*.

[54] Apte S, Sanders DA (2010). Effects of retroviral envelope-protein cleavage upon trafficking, incorporation, and membrane fusion. *Virology*.

[55] Grange MP, Blot V, Delamarre L (2000). Identification of two intracellular mechanisms leading to reduced expression of oncoretrovirus envelope glycoproteins at the cell surface. *Journal of Virology*.

[56] Ilinskaya A, Heidecker G, Derse D (2010). Opposing effects of a tyrosine-based sorting motif and a PDZ-binding motif regulate human T-lymphotropic virus type 1 envelope trafficking. *Journal of Virology*.

[57] Sandrin V, Muriaux D, Darlix JL, Cosset FL (2004). Intracellular trafficking of Gag and Env proteins and their interactions modulate pseudotyping of retroviruses. *Journal of Virology*.

[58] Berlioz-Torrent C, Shacklett BL, Erdtmann L (1999). Interactions of the cytoplasmic domains of human and simian retroviral transmembrane proteins with components of the clathrin adaptor complexes modulate intracellular and cell surface expression of envelope glycoproteins. *Journal of Virology*.

[59] Wyss S, Berlioz-Torrent C, Boge M (2001). The highly conserved C-terminal dileucine motif in the cytosolic domain of the human immunodeficiency virus type 1 envelope glycoprotein is critical for its association with the AP-1 clathrin adapter. *Journal of Virology*.

[60] Miranda LR, Schaefer BC, Kupfer A, Hu Z, Franzusoff A (2002). Cell surface expression of the HIV-1 envelope glycoproteins is directed from intracellular CTLA-4-containing regulated secretory granules. *Proceedings of the National Academy of Sciences of the United States of America*.

[61] Boge M, Wyss S, Bonifacino JS, Thali M (1998). A membrane-proximal tyrosine-based signal mediates internalization of the HIV-1 envelope glycoprotein via interaction with the AP-2 clathrin adaptor. *Journal of Biological Chemistry*.

[62] Ohno H, Aguilar RC, Fournier MC, Hennecke S, Cosson P, Bonifacino JS (1997). Interaction of endocytic signals from the HIV-1 envelope glycoprotein complex with members of the adaptor medium chain family. *Virology*.

[63] Egan MA, Carruth LM, Rowell JF, Yu X, Siliciano RF (1996). Human immunodeficiency virus type 1 envelope protein endocytosis mediated by a highly conserved intrinsic internalization signal in the cytoplasmic domain of gp41 is suppressed in the presence of the Pr55(gag) precursor protein. *Journal of Virology*.

[64] Rowell JF, Stanhope PE, Siliciano RF (1995). Endocytosis of endogenously synthesized HIV-1 envelope protein: mechanism and role in processing for association with class II MHC. *Journal of Immunology*.

[65] Blot V, Lopez-Vergès S, Breton M, Pique C, Berlioz-Torrent C, Grange MP (2006). The conserved dileucine- and tyrosine-based motifs in MLV and MPMV envelope glycoproteins are both important to regulate a common Env intracellular trafficking. *Retrovirology*.

[66] Bouard D, Sandrin V, Boson B (2007). An acidic cluster of the cytoplasmic tail of the RD114 virus glycoprotein controls assembly of retroviral envelopes. *Traffic*.

[67] Cosset FL, Takeuchi Y, Battini JL, Weiss RA, Collins MKL (1995). High-titer packaging cells producing recombinant retroviruses resistant to human serum. *Journal of Virology*.

[68] Sandrin V, Boson B, Salmon P (2002). Lentiviral vectors pseudotyped with a modified RD114 envelope glycoprotein show increased stability in sera and augmented transduction of primary lymphocytes and CD34^+^ cells derived from human and nonhuman primates. *Blood*.

[69] Takeuchi Y, Cosset FLC, Lachmann PJ, Okada H, Weiss RA, Collins MKL (1994). Type C retrovirus inactivation by human complement is determined by both the viral genome and the producer cell. *Journal of Virology*.

[70] Johnson MC (2011). Mechanisms for env glycoprotein acquisition by retroviruses. *AIDS Research and Human Retroviruses*.

[71] Bartosch B, Dubuisson J, Cosset FL (2003). Infectious hepatitis C virus pseudo-particles containing functional E1-E2 envelope protein complexes. *Journal of Experimental Medicine*.

[72] Christodoulopoulos I, Cannon PM (2001). Sequences in the cytoplasmic tail of the gibbon ape leukemia virus envelope protein that prevent its incorporation into lentivirus vectors. *Journal of Virology*.

[73] Hofmann H, Hattermann K, Marzi A (2004). S protein of severe acute respiratory syndrome-associated coronavirus mediates entry into hepatoma cell lines and is targeted by neutralizing antibodies in infected patients. *Journal of Virology*.

[74] Kobinger GP, Deng S, Louboutin JP (2004). Transduction of human islets with pseudotyped lentiviral vectors. *Human Gene Therapy*.

[75] Kobinger GP, Weiner DJ, Yu QC, Wilson JM (2001). Filovirus-pseudotyped lentiviral vector can efficiently and stably transduce airway epithelia in vivo. *Nature Biotechnology*.

[76] Kumar M, Bradow BP, Zimmerberg J (2003). Large-scale production of pseudotyped lentiviral vectors using baculovirus GP64. *Human Gene Therapy*.

[77] Landau NR, Page KA, Littman DR (1991). Pseudotyping with human T-cell leukemia virus type I broadens the human immunodeficiency virus host range. *Journal of Virology*.

[78] Lewis BC, Chinnasamy N, Morgan RA, Varmus HE (2001). Development of an avian leukosis-sarcoma virus subgroup a pseudotyped lentiviral vector. *Journal of Virology*.

[79] Liu SL, Halbert CL, Miller AD (2004). Jaagsiekte sheep retrovirus envelope efficiently pseudotypes human immunodeficiency virus type 1-based lentiviral vectors. *Journal of Virology*.

[80] Mochizuki H, Schwartz JP, Tanaka K, Brady RO, Reiser J (1998). High-titer human immunodeficiency virus type 1-based vector systems for gene delivery into nondividing cells. *Journal of Virology*.

[81] Morizono M, Bristol G, Xie YM, Kung SKP, Chen ISY (2001). Antibody-directed targeting of retroviral vectors via cell surface antigens. *Journal of Virology*.

[82] Naldini L, Blömer U, Gallay P (1996). In vivo gene delivery and stable transduction of nondividing cells by a lentiviral vector. *Science*.

[83] Reiser J, Harmison G, Kluepfel-Stahl S, Brady RO, Karlsson S, Schubert M (1996). Transduction of nondividing cells using pseudotyped defective high-titer HIV type 1 particles. *Proceedings of the National Academy of Sciences of the United States of America*.

[84] Zeilfelder U, Bosch V (2001). Properties of wild-type, C-terminally truncated, and chimeric maedi-visna virus glycoprotein and putative pseudotyping of retroviral vector particles. *Journal of Virology*.

[85] Chertova E, Chertov O, Coren LV (2006). Proteomic and biochemical analysis of purified human immunodeficiency virus type 1 produced from infected monocyte-derived macrophages. *Journal of Virology*.

[86] Hammarstedt M, Garoff H (2004). Passive and active inclusion of host proteins in human immunodeficiency virus type 1 Gag particles during budding at the plasma membrane. *Journal of Virology*.

[87] Hammarstedt M, Wallengren K, Pedersen KW, Roos N, Garoff H (2000). Minimal exclusion of plasma membrane proteins during retroviral envelope formation. *Proceedings of the National Academy of Sciences of the United States of America*.

[88] Arthur LO, Bess JW, Sowder RC (1992). Cellular proteins bound to immunodeficiency viruses: implications for pathogenesis and vaccines. *Science*.

[89] Ott DE (2008). Cellular proteins detected in HIV-1. *Reviews of Medical Virology*.

[90] Chen SSL, Ferrante AA, Terwilliger EF (1996). Characterization of an envelope mutant of HIV-1 that interferes with viral infectivity. *Virology*.

[91] Freed EO, Martin MA (1996). Domains of the human immunodeficiency virus type 1 matrix and gp41 cytoplasmic tail required for envelope incorporation into virions. *Journal of Virology*.

[92] Freed EO, Martin MA (1995). Virion incorporation of envelope glycoproteins with long but not short cytoplasmic tails is blocked by specific, single amino acid substitutions in the human immunodeficiency virus type 1 matrix. *Journal of Virology*.

[93] Murakami T, Freed EO (2000). The long cytoplasmic tail of gp41 is required in a cell type-dependent manner for HIV-1 envelope glycoprotein incorporation into virions. *Proceedings of the National Academy of Sciences of the United States of America*.

[94] Wilk T, Pfeiffer T, Bosch V (1992). Retained in vitro infectivity and cytopathogenicity of HIV-1 despite truncation of the C-terminal tail of the env gene product. *Virology*.

[95] Dubay JW, Roberts SJ, Hahn BH, Hunter E (1992). Truncation of the human immunodeficiency virus type 1 transmembrane glycoprotein cytoplasmic domain blocks virus infectivity. *Journal of Virology*.

[96] Gabuzda DH, Lever A, Terwilliger E, Sodroski J (1992). Effects of deletions in the cytoplasmic domain on biological functions of human immunodeficiency virus type 1 envelope glycoproteins. *Journal of Virology*.

[97] Iwatani Y, Ueno T, Nishimura A (2001). Modification of virus infectivity by cytoplasmic tail of HIV-1 TM protein. *Virus Research*.

[98] Murakami T, Freed EO (2000). Genetic evidence for an interaction between human immunodeficiency virus type 1 matrix and *α*-helix 2 of the gp41 cytoplasmic tail. *Journal of Virology*.

[99] Piller SC, Dubay JW, Derdeyn CA, Hunter E (2000). Mutational analysis of conserved domains within the cytoplasmic tail of gp41 from human immunodeficiency virus type 1: effects on glycoprotein incorporation and infectivity. *Journal of Virology*.

[100] Yu X, Yuan X, McLane MF, Lee TH, Essex M (1993). Mutations in the cytoplasmic domain of human immunodeficiency virus type 1 transmembrane protein impair the incorporation of Env proteins into mature virions. *Journal of Virology*.

[101] Dorfman T, Mammano F, Haseltine WA, Gottlinger HG (1994). Role of the matrix protein in the virion association of the human immunodeficiency virus type 1 envelope glycoprotein. *Journal of Virology*.

[102] Yu X, Yuan X, Matsuda Z, Lee TH, Essex M (1992). The matrix protein of human immunodeficiency virus type 1 is required for incorporation of viral envelope protein into mature virions. *Journal of Virology*.

[103] Lodge R, Gottlinger H, Gabuzda D, Cohen EA, Lemay G (1994). The intracytoplasmic domain of gp41 mediates polarized budding of human immunodeficiency virus type 1 in MDCK cells. *Journal of Virology*.

[104] Lodge R, Lalonde JP, Lemay G, Cohen EA (1997). The membrane-proximal intracytoplasmic tyrosine residue of HIV-1 envelope glycoprotein is critical for basolateral targeting of viral budding in MDCK cells. *The EMBO Journal*.

[105] Owens RJ, Compans RW (1989). Expression of the human immunodeficiency virus envelope glycoprotein is restricted to basolateral surfaces of polarized epithelial cells. *Journal of Virology*.

[106] Owens RJ, Dubay JW, Hunter E, Compans RW (1991). Human immunodeficiency virus envelope protein determines the site of virus release in polarized epithelial cells. *Proceedings of the National Academy of Sciences of the United States of America*.

[107] Deschambeault J, Lalonde JP, Cervantes-Acosta G, Lodge R, Cohen EA, Lemay G (1999). Polarized human immunodeficiency virus budding in lymphocytes involves a tyrosine-based signal and favors cell-to-cell viral transmission. *Journal of Virology*.

[108] Lodge R, Delamarre L, Lalonde JP (1997). Two distinct oncornaviruses harbor an intracytoplasmic tyrosine-based basolateral targeting signal in their viral envelope glycoprotein. *Journal of Virology*.

[109] Weclewicz K, Ekström M, Kristensson K, Garoff H (1998). Specific interactions between retrovirus Env and Gag proteins in rat neurons. *Journal of Virology*.

[110] Lucas TM, Lyddon TD, Grosse SA, Johnson MC (2010). Two distinct mechanisms regulate recruitment of murine leukemia virus envelope protein to retroviral assembly sites. *Virology*.

[111] Jorgenson RL, Vogt VM, Johnson MC (2009). Foreign glycoproteins can be actively recruited to virus assembly sites during pseudotyping. *Journal of Virology*.

[112] Cosson P (1996). Direct interaction between the envelope and matrix proteins of HIV-1. *The EMBO Journal*.

[113] Manrique JM, Affranchino JL, González SA (2008). In vitro binding of simian immunodeficiency virus matrix protein to the cytoplasmic domain of the envelope glycoprotein. *Virology*.

[114] Hourioux C, Brand D, Sizaret PY (2000). Identification of the glycoprotein 41(TM) cytoplasmic tail domains of human immunodeficiency virus type 1 that interact with Pr55(Gag) particles. *AIDS Research and Human Retroviruses*.

[115] Davis MR, Jiang J, Zhou J, Freed EO, Aiken C (2006). A mutation in the human immunodeficiency virus type 1 Gag protein destabilizes the interaction of the envelope protein subunits gp120 and gp41. *Journal of Virology*.

[116] Jiang J, Aiken C (2007). Maturation-dependent human immunodeficiency virus type 1 particle fusion requires a carboxyl-terminal region of the gp41 cytoplasmic tail. *Journal of Virology*.

[117] Blot G, Janvier K, Le Panse S, Benarous R, Berlioz-Torrent C (2003). Targeting of the human immunodeficiency virus type 1 envelope to the trans-Golgi network through binding to TIP47 is required for Env incorporation into virions and infectivity. *Journal of Virology*.

[118] Lopez-Vergès S, Camus G, Blot G, Beauvoir R, Benarous R, Berlioz-Torrent C (2006). Tail-interacting protein TIP47 is a connector between Gag and Env and is required for Env incorporation into HIV-1 virions. *Proceedings of the National Academy of Sciences of the United States of America*.

[119] Bauby H, Lopez-Vergès S, Hoeffel G (2010). TIP47 is required for the production of infectious HIV-1 particles from primary macrophages. *Traffic*.

[120] Blot V, Delamarre L, Perugi F (2004). Human Dlg protein binds to the envelope glycoproteins of human T-cell leukemia virus type 1 and regulates envelope mediated cell-cell fusion in T lymphocytes. *Journal of Cell Science*.

[121] Perugi F, Muriaux D, Ramirez BC (2009). Human discs large is a new negative regulator of human immunodeficiency virus-1 infectivity. *Molecular Biology of the Cell*.

[122] Evans DT, Tillman KC, Desrosiers RC (2002). Envelope glycoprotein cytoplasmic domains from diverse lentiviruses interact with the prenylated rab acceptor. *Journal of Virology*.

[123] Blancou P, Evans DT, Desrosiers RC (2005). PRA1 co-localizes with envelope but does not influence primate lentivirus production, infectivity or envelope incorporation. *Journal of General Virology*.

[124] Emerson V, Holtkotte D, Pfeiffer T (2010). Identification of the cellular prohibitin 1/prohibitin 2 heterodimer as an interaction partner of the C-terminal cytoplasmic domain of the HIV-1 glycoprotein. *Journal of Virology*.

[125] Merkwirth C, Langer T (2009). Prohibitin function within mitochondria: essential roles for cell proliferation and cristae morphogenesis. *Biochimica et Biophysica Acta*.

[126] Mishra S, Ande SR, Nyomba BLG (2010). The role of prohibitin in cell signaling. *FEBS Journal*.

[127] Blot G, Lopez-Vergès S, Treand C (2006). Luman, a new partner of HIV-1 TMgp41, interferes with tat-mediated transcription of the HIV-1 LTR. *Journal of Molecular Biology*.

[128] Zhang H, Wang L, Kao S (1999). Functional interaction between the cytoplasmic leucine-zipper domain of HIV-1 gp41 and p115-RhoGEF. *Current Biology*.

[129] Miller MA, Mietzner TA, Cloyd MW, Robey WG, Montelaro RC (1993). Identification of a calmodulin-binding and inhibitory peptide domain in the HIV-1 transmembrane glycoprotein. *AIDS Research and Human Retroviruses*.

[130] Srinivas SK, Srinivas RV, Anantharamaiah GM, Compans RW, Segrest JP (1993). Cytosolic domain of the human immunodeficiency virus envelope glycoproteins binds to calmodulin and inhibits calmodulin-regulated proteins. *Journal of Biological Chemistry*.

[131] Tencza SB, Mietzner TA, Montelaro RC (1997). Calmodulin-binding function of LLP segments from the HIV type 1 transmembrane protein is conserved among natural sequence variants. *AIDS Research and Human Retroviruses*.

[132] Tencza SB, Miller MA, Islam K, Mietzner TA, Montelaro RC (1995). Effect of amino acid substitutions on calmodulin binding and cytolytic properties of the LLP-1 peptide segment of human immunodeficiency virus type 1 transmembrane protein. *Journal of Virology*.

[133] Drees F, Pokutta S, Yamada S, Nelson WJ, Weis WI (2005). *α*-catenin is a molecular switch that binds E-cadherin-*β*-catenin and regulates actin-filament assembly. *Cell*.

[134] Kim EM, Lee KH, Kim JW (1999). The cytoplasmic domain of HIV-1 gp41 interacts with the carboxyl-terminal region of *α*-catenin. *Molecules and Cells*.

[135] Jong TK, Eun MK, Kyoung HL, Choi JE, Jhun BH, Jung WK (2002). Leucine zipper domain of HIV-1 gp41 interacted specifically with *α*-catenin. *Biochemical and Biophysical Research Communications*.

[136] Munro S (2003). Lipid rafts: elusive or illusive?. *Cell*.

[137] Simons K, Gerl MJ (2010). Revitalizing membrane rafts: new tools and insights. *Nature Reviews Molecular Cell Biology*.

[138] Brown DA, London E (2000). Structure and function of sphingolipid- and cholesterol-rich membrane rafts. *Journal of Biological Chemistry*.

[139] Simons K, Toomre D (2000). Lipid rafts and signal transduction. *Nature Reviews Molecular Cell Biology*.

[140] Lim KI, Narayan S, Young JAT, Yin J (2004). Effects of lipid rafts on dynamics of retroviral entry and trafficking: quantitative analysis. *Biotechnology and Bioengineering*.

[141] Narayan S, Barnard RJO, Young JAT (2003). Two retroviral entry pathways distinguished by lipid raft association of the viral receptor and differences in viral infectivity. *Journal of Virology*.

[142] Nguyen DH, Hildreth JEK (2000). Evidence for budding of human immunodeficiency virus type 1 selectively from glycolipid-enriched membrane lipid rafts. *Journal of Virology*.

[143] Ono A, Freed EO (2001). Plasma membrane rafts play a critical role in HIV-1 assembly and release. *Proceedings of the National Academy of Sciences of the United States of America*.

[144] Pickl WF, Pimentel-Muiñios FX, Seed B (2001). Lipid rafts and pseudotyping. *Journal of Virology*.

[145] Waheed AA, Freed EO (2009). Lipids and membrane microdomains in HIV-1 replication. *Virus Research*.

[146] Waheed AA, Freed EO (2010). The role of lipids in retrovirus replication. *Viruses*.

[147] Briggs JAG, Wilk T, Fuller SD (2003). Do lipid rafts mediate virus assembly and pseudotyping?. *Journal of General Virology*.

[148] Graham DRM, Chertova E, Hilburn JM, Arthur LO, Hildreth JEK (2003). Cholesterol depletion of human immunodeficiency virus type 1 and simian immunodeficiency virus with *β*-cyclodextrin inactivates and permeabilizes the virions: evidence for virion-associated lipid rafts. *Journal of Virology*.

[149] Lindwasser OW, Resh MD (2001). Multimerization of human immunodeficiency virus type 1 Gag promotes its localization to barges, raft-like membrane microdomains. *Journal of Virology*.

[150] Rousso I, Mixon MB, Chen BK, Kim PS (2000). Palmitoylation of the HIV-1 envelope glycoprotein is critical for viral infectivity. *Proceedings of the National Academy of Sciences of the United States of America*.

[151] Hemler ME (2005). Tetraspanin functions and associated microdomains. *Nature Reviews Molecular Cell Biology*.

[152] Jolly C, Sattentau QJ (2007). Human immunodeficiency virus type 1 assembly, budding, and cell-cell spread in T cells take place in tetraspanin-enriched plasma membrane domains. *Journal of Virology*.

[153] Krementsov DN, Rassam P, Margeat E (2010). HIV-1 assembly differentially alters dynamics and partitioning of tetraspanins and raft components. *Traffic*.

[154] Nydegger S, Khurana S, Krementsov DN, Foti M, Thali M (2006). Mapping of tetraspanin-enriched microdomains that can function as gateways for HIV-1. *Journal of Cell Biology*.

[155] Martin F, Roth DM, Jans DA (2005). Tetraspanins in viral infections: a fundamental role in viral biology?. *Journal of Virology*.

[156] Khurana S, Krementsov DN, de Parseval A, Elder JH, Foti M, Thali M (2007). Human immunodeficiency virus type 1 and influenza virus exit via different membrane microdomains. *Journal of Virology*.

[157] Hogue IB, Grover JR, Soheilian F, Nagashima K, Ono A (2011). Gag induces the coalescence of clustered lipid rafts and tetraspanin-enriched microdomains at HIV-1 assembly sites on the plasma membrane. *Journal of Virology*.

[158] Leung K, Kim JO, Ganesh L, Kabat J, Schwartz O, Nabel GJ (2008). HIV-1 assembly: viral glycoproteins segregate quantally to lipid rafts that associate individually with HIV-1 capsids and virions. *Cell Host and Microbe*.

[159] Nejmeddine M, Bangham CRM (2010). The HTLV-1 virological synapse. *Viruses*.

[160] Sato H, Orenstein J, Dimitrov D, Martin M (1992). Cell-to-cell spread of HIV-1 occurs within minutes and may not involve the participation of virus particles. *Virology*.

[161] Igakura T, Stinchcombe JC, Goon PKC (2003). Spread of HTLV-I between lymphocytes by virus-induced polarization of the cytoskeleton. *Science*.

[162] Sattentau QJ (2010). Cell-to-cell spread of retroviruses. *Viruses*.

[163] Sowinski S, Jolly C, Berninghausen O (2008). Membrane nanotubes physically connect T cells over long distances presenting a novel route for HIV-1 transmission. *Nature Cell Biology*.

[164] Pais-Correia AM, Sachse M, Guadagnini S (2010). Biofilm-like extracellular viral assemblies mediate HTLV-1 cell-to-cell transmission at virological synapses. *Nature Medicine*.

[165] Sherer NM, Jin J, Mothes W (2010). Directional spread of surface-associated retroviruses regulated by differential virus-cell interactions. *Journal of Virology*.

[166] Sherer NM, Lehmann MJ, Jimenez-Soto LF, Horensavitz C, Pypaert M, Mothes W (2007). Retroviruses can establish filopodial bridges for efficient cell-to-cell transmission. *Nature Cell Biology*.

[167] Jin J, Li F, Mothes W (2011). Viral determinants of polarized assembly for the murine leukemia virus. *Journal of Virology*.

[168] Jin J, Sherer NM, Heidecker G, Derse D, Mothes W (2009). Assembly of the murine leukemia virus is directed towards sites of cell-cell contact. *PLoS Biology*.

